# Direct measurement of CTDI_w_ on helical CT scans

**DOI:** 10.1002/acm2.13761

**Published:** 2022-10-06

**Authors:** Kyle D. DePew, Robert C. Boggs, Michael V. Yester, Gary T. Barnes

**Affiliations:** ^1^ Department of Radiology University of Alabama at Birmingham Birmingham Alabama USA

**Keywords:** computed tomography, CTDI, CTDI_w_ helical acquisition

## Abstract

**Purpose:**

Medical physics computed tomography (CT) practice involves measurements to determine CTDI_vol_ on representative clinical CT protocols. In current practice the majority of CT exams employ helical scans. To determine CTDI_vol_ for a helical scan, one measures CTDI_w_ with an axial scan, then divides by the pitch. Problems arise in CT units where one is unable to select an axial scan with the same detector configuration and pre‐patient (bowtie) filtration that is employed on the helical scan. Presented is a method to measure CTDI_w_ on helical scans.

**Methods:**

The body and head CTDI phantoms were supported on the gantry shroud with brackets attached to the phantom. The phantom is above the tabletop and remains stationary during helical scans as the table moves beneath the phantom. With the phantom stationary, the CTDI_w_ associated with head and body helical scans was measured. CTDI_w_ was also measured for head and body axial scans with the same pre‐patient filtrations and detector configurations.

**Results:**

For both the head and body CTDI phantom the agreement between the axial and helical CTDI_w_ measurements was <1.5%.

**Conclusions:**

Body and head CTDI_w_ and CTDI_vol_ can be directly measured by employing helical scans with the method in this paper

## 1 INTRODUCTION

Volumetric computed tomography dose index or CTDI_vol_ is routinely determined by medical physicists for clinical scan protocols during CT scanner acceptance testing, annual performance audits, and ACR CT Accreditation submission data acquisition.^1^, ^2^ This is accomplished by centering the CTDI phantom in the CT gantry, measuring CTDI_w_ with an axial scan (keeping the phantom stationary), and dividing by the pitch.

In present day clinical practice, many, if not the majority, of the CT exams employ helical scan protocols. On some CT scanners, for example, Siemens Force and Drive models, axial scans with the same detector configuration and pre‐patient filtration (bowtie filter) employed on helical protocols are not available. Measuring CTDI_w_ and determining CTDI_vol_ on these scanners is problematic. One approach to the problem is to obtain a service key from the manufacturer which permits one to make the desired axial scans and measure the CTDI_w_ associated with the helical scan protocol of interest. However, service keys are difficult to obtain, and are not always available. Obtaining service keys is also not feasible for the consulting medical physicist who has a number of different scanners to support and a limited time window to make his/her measurements.

An alternative approach to the problem recently described in the literature is to measure CTDI_vol_ by scanning the CTDI phantom helically.^3^ Although eminently practical and providing consistent results appropriate for quality control (QC) checks, there are systematic scatter contribution differences between this approach and the definition of CTDI_vol_. Presented is a method to determine CTDI_vol_ consistent with its definition on helical scan protocols by directly measuring CTDI_w_ on the helical scan.

## 2 MATERIALS AND METHODS

**FIGURE 1:**
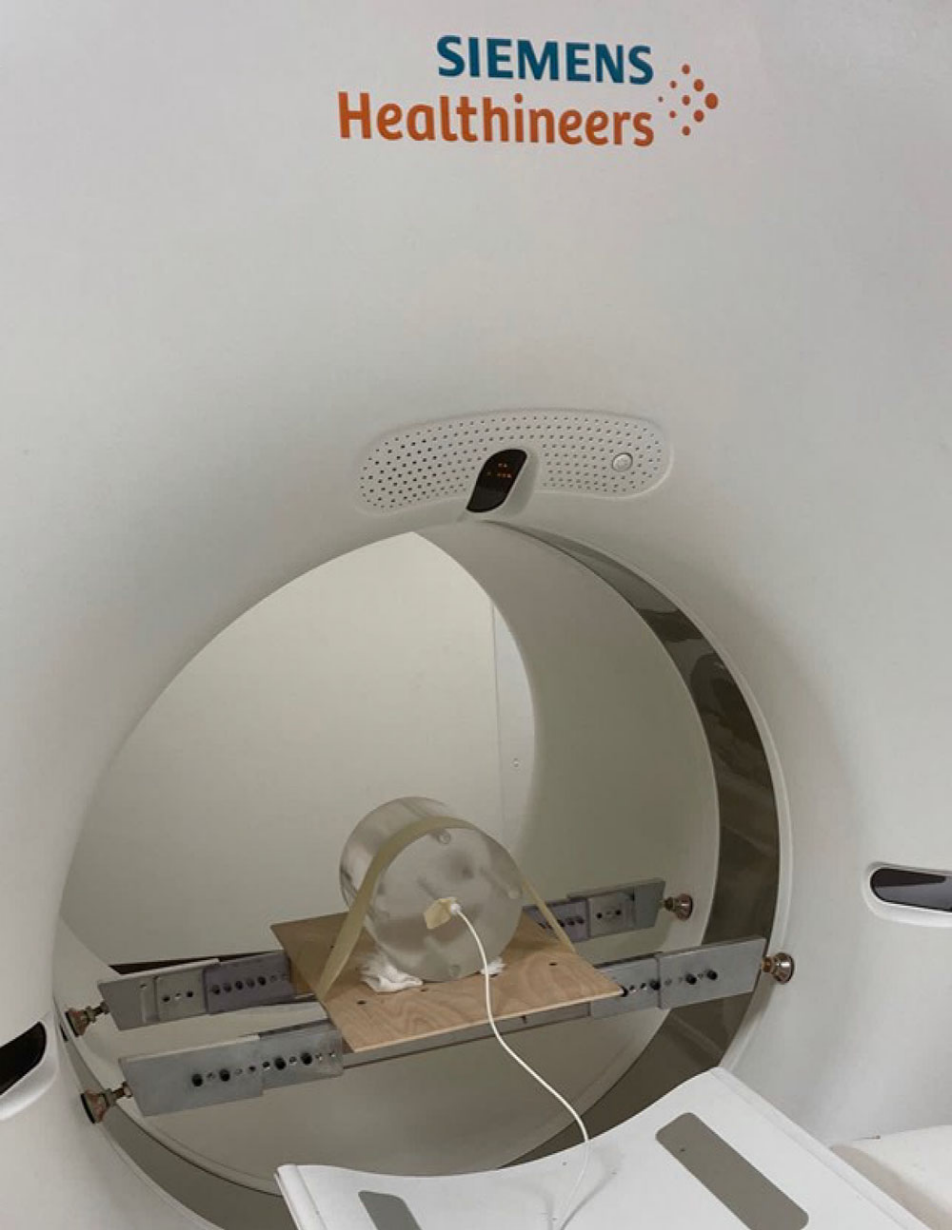
Positioning of body phantom with brackets. Shown is the body CTDI phantom suspended on the GE Discovery CT gantry shroud and above the scanner tabletop with brackets. The brackets were originally developed at UAB 20+ years ago to facilitate long helical exposure times for CT environmental radiation surveys.

The body CTDI phantom was modified by the addition of brackets to the sides of the phantom. The brackets (Support Bracket, Model 007‐01, CIRS, Norfolk, VA), support the phantom on the CT gantry shroud with the tabletop below. One attaches the brackets to the phantom, centers the phantom in the gantry by adjusting the tabletop height and position, adjusts the lengths of the brackets so that they are in contact with the gantry shroud, and lowers the tabletop 1 to 2 cm below the phantom. As shown in Figure 1 the body CTDI phantom is supported by the brackets and gantry shroud. These brackets have been used on CTs at our institution for more than two decades and have never damaged the gantry shroud. Even though the CTDI body phantom itself is large, the CT gantry is constructed to withstand large forces, and has no problem with the apparatus. Additionally, the rubber feet prevent the brackets from scratching the shroud.

During a helical scan the tabletop moves and the phantom remains stationary. One measures the surface and axis exposures associated with the helical scan. The measured exposures are divided by the number of gantry rotations during the scan, *N*_rot_, to determine values for one rotation, and CTDI_w_ is calculated. The number of gantry rotations is given by

(1) $$N_{\mathrm{rot}}=\mathrm{Helical}\mathrm{scan}\mathrm{distance}/\left(\mathrm{Table}\mathrm{movement}\mathrm{per}\mathrm{rotation}\right)$$

**FIGURE 2:**
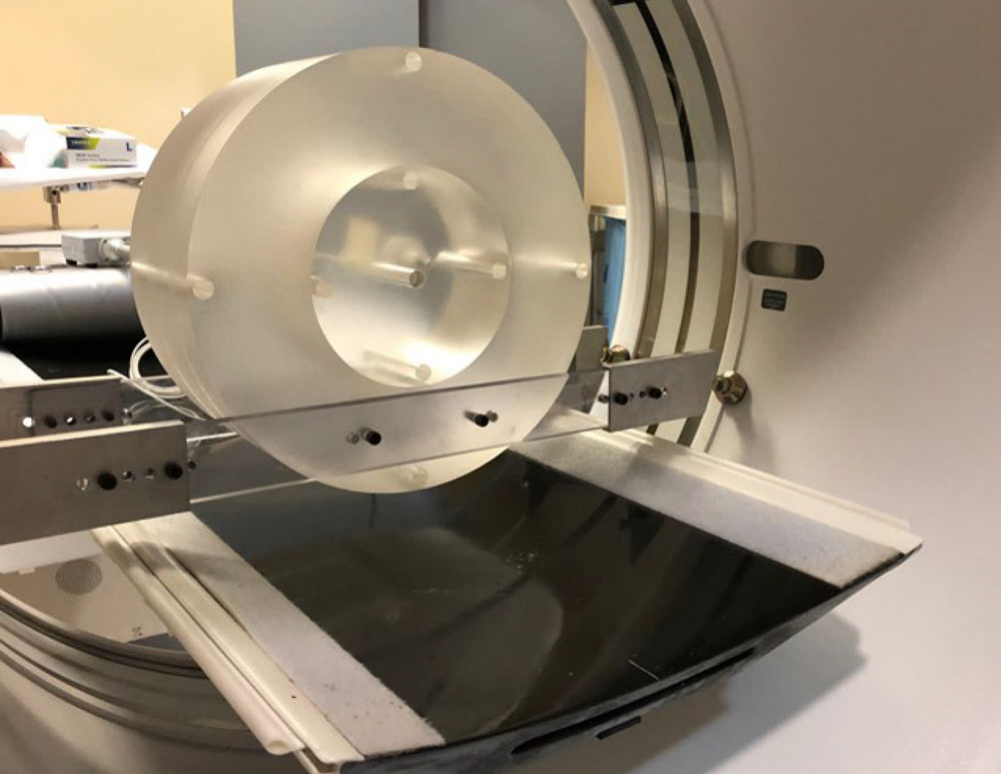
Positioning of head phantom on lauan board. Shown is the head CTDI phantom suspended and centered in the CT gantry. The phantom is taped to a 1/4″ thick lauan board. The lauan is attached to and supported by brackets which in turn are supported by the CT gantry shroud. The brackets are the same as the brackets used to suspend the body CTDI phantom in Figure 1.

The helical scan distance is the distance the tabletop travels while the x‐ray beam is on and was determined from the technical information associated with the scan or by dividing the displayed DLP (dose‐length‐product) by the displayed CTDI_vol_. Alternatively, the number of rotations can be determined by dividing chamber exposure time by the rotation time. Exposure per rotation may be calculated as:

(2) $$X_{\mathrm{rot}}=X_{\mathrm{measured}}/N_{\mathrm{rot}}$$

where *X*_rot_ is defined as the exposure per rotation, *X*_measured_ is defined as the exposure measured during an entire acquisition, and *N*_rot_ is the number of rotations in the acquisition. This method was first used in the acceptance test of a recently installed Siemens Somatom Drive CT scanner that did not have axial scan protocols with the same detector configurations as those employed in the helical protocols. To confirm the validity of the method, CTDI_w_ and CTDI_vol_ were measured with the adult body and head CTDI phantom for helical and axial scans on a GE Discovery CT scanner, employing the same detector configuration and technical factors. Shown in Figure 2 is the head CTDI phantom positioned in the center of the Siemens gantry for a helical (and axial) CTDI_w_ acquisition. The head holder is removed and phantom is taped to a 6.35 mm (1/4″) thick lauan plywood board attached to the same brackets that are employed for the body CTDI_w_ measurements.

In this study, for axial scans, if the table increment between rotations is the same as the beamwidth, the pitch is defined as 1. If the table increment between rotations is greater than the beamwidth, the pitch is greater than 1. Likewise, if it is less, the pitch is less than 1.

## 3 RESULTS

As noted above this method was first used on a Siemens Somatom Drive CT scanner. The difference between the measured CTDI_vol_ and the value displayed by the scanner is 2% for the head and 5% for the body. Table 1 lists the helical and axial body CTDI acquisitions made on the GE Discovery CT. The measured CTDI_w_ values acquired with the two scan protocols agree to within 1%. Table 2 lists helical (two scans with different pitches) and axial head CTDI acquisitions made on the GE Discovery unit. The coefficient of variation of the three CTDI_w_ measurements is 0.7%. In addition, the axial head CTDI_w_ was measured with the head phantom supported by the GE head holder rather than by the lauan board. The axial measurement with the head holder agreed with the suspended phantom measurement to within 1%. Table 3 lists helical and axial head CTDI acquisitions made on a Siemens Somatom Drive CT scanner. The agreement of the two head CTDI_w_ values is within 1.5%. We would therefore recommend between 5 and 10 rotations to minimize error.

**TABLE 1:** Comparison of axial and helical CTDI_w_ acquisitions and CTDI_vol_ determinations on GE Discovery CT scanner with body (32 cm diameter) CTDI phantom

**Scan FOV**	**Large body**	**Large body**
X‐ray tube potential (kV)	120	120
X‐ray tube current (mA)	250	250
Rotation time (s)	0.6	0.6
Axial (A) or helical (H)	H	A
Pitch	0.984	1.0
Number of rotations	5.92	1.0
Measured CTDI_w_ (mGy)	11.06	11.11
Measured CTDI_vol_ (mGy)	11.24	—
Displayed CTDI_vol_ (mGy)	12.22	—
Displayed/measured CTDI_vol_	1.09	—

**TABLE 2:** Comparison of helical and axial CTDI_w_ acquisitions and CTDI_vol_ determinations on GE Discovery CT scanner with head (16 cm diameter) CTDI phantom

**Scan FOV**	**Head**	**Head**	**Head**
X‐ray tube potential (kV)	120	120	120
X‐ray tube current (mA)	250	250	250
Rotation time (s)	1.0	1.0	1.0
Axial (A) or helical (H)	H	H	A
Pitch	0.531	0.969	1.0
Number of rotations	10.064	5.587	1.0
Measured CTDI_w_ (mGy)	46.13	45.70	46.38
Measured CTDI_vol_ (mGy)	86.88	47.19	—
Displayed CTDI_vol_ (mGy)	93.95	51.52	—
Displayed/measured CTDI_vol_	1.081	1.092	—

**TABLE 3:** Comparison of axial and helical CTDIw acquisitions and CTDIvol determinations on Siemens Somatom Drive CT scanner with head (16 cm diameter) CTDI phantom

**Scan FOV**	**30 cm**	**30 cm**
X‐ray tube potential (kV)	140	140
Effective mAs	190	152
X‐ray tube current (mA)	152	152
Rotation time (s)	1.0	1.0
Axial (A) or helical (H)	H	A
Pitch	0.8	1.0
Number of rotations	13.85	1.0
Measured CTDI_w_ (mGy)	40.08	40.68
Measured CTDI_vol_ (mGy)	50.10	—
Displayed CTDI_vol_ (mGy)	46.77	—
Displayed/measured CTDI_vol_	0.93	—

## 4 DISCUSSION

The Siemens Somatom Drive CTDI_w_ head axial and helical results match within 1.5%. The results for the GE Discovery body axial and helical CTDI_w_ are within 0.5%. The good agreement for the axial and helical results validates the suspended phantom method. This is not unexpected as the CTDI phantom is stationary for both the axial and helical scan protocols. The only differences are: (1) the tabletop moves in one case and not the other; (2) exposure times are longer for helical than for axial scans; and (3) in helical scans the dosimeter reading is for several rotations while in axial scans the dosimeter reading is for one rotation (one divides the helical reading by the number of rotations to determine the result for one rotation).

Critical to the direct helical CTDI_w_ measurements is determining the number of rotations associated with the helical scan. For the measurements presented, this was determined by dividing the scan distance by the tabletop movement per rotation. Over‐ranging occurs at the start and end of helical scans, and it is important to note that the scan distance is greater than the distance spanned by the reconstructed images. An alternative approach to determine the number of rotations is to measure the x‐ray exposure time with the ion chamber or separate sensor during the helical scan and divide the exposure time by the rotation time.

Both the GE and Siemens CT scanners on which the above measurements were made are ACR accredited. For the GE scanner, axial dosimetry measures were submitted. For the Siemens scanner, the suspended CTDI phantom method was employed and the helical dosimetry readings divided by the number of rotations were submitted.

Leon et al. propose a helically acquired CTDI_vol_ as an alternative to making axial CTDI_w_ measurements to determine CTDI_vol_, and that its use is appropriate for QC purposes.^3^ The helically acquired CTDI_vol_ is much easier and more time‐efficient than CTDI_vol_ determined with the suspended phantom method. However, as noted by Leon et al., it is not a perfect match for the traditionally determined CTDI_vol_ measured with the CTDI phantom stationary, as the scatter contributions are different. This is due to the fact that the CTDI phantom is bounded by air, which does not attenuate and scatter radiation to the same extent as acrylic will. As the phantom moves out of the beam, less radiation is scattered back into the phantom and the detector than would be for a corresponding slice in the middle of the CTDI phantom. In our practice, on scanners where axial scans with the same pre‐patient filtration and detector configurations employed on helical protocols are not available, we plan to measure initially both the helically acquired CTDI_vol_ and the suspended phantom measured CTDI_vol_, and develop a correction factor. Subsequently for routine QC we will measure the helically acquired CTDI_vol_ and use the correction factor to make the measurement more representative of the traditional CTDI_vol_ determination.

Our method may also be useful in the measurement of cone‐beam CBDI as well (defined as in ref. [4]). Mobile cone‐beam units may also move during acquisition,^5^ meaning an adaptation of this method may be useful.

## 5 CONCLUSIONS

The good agreement of axial and helical results for the GE Discovery scanner (CTDI body and head phantoms) and for the Siemens Somatom Drive scanner (CTDI head phantom) demonstrates that the suspended phantom method is viable and allows one to directly measure CTDI_w_ and CTDI_vol_ with helical scans.

## CONFLICT OF INTEREST

The authors declare no conflict of interest.
